# Post-COVID Metabolic Fallout: A Growing Threat of New-Onset and Exacerbated Diabetes

**DOI:** 10.3390/biomedicines13061482

**Published:** 2025-06-16

**Authors:** Shaghayegh Hemat Jouy, Harry Tonchev, Sarah M. Mostafa, Abeer M. Mahmoud

**Affiliations:** 1 Department of Exercise Physiology, Faculty of Physical Education and Sport Sciences, Central Tehran Branch, Islamic Azad University, Tehran 11369, Iran; shaghayegh.hematjouy@gmail.com; 2 Division of Endocrinology, Diabetes, and Metabolism, Department of Medicine, College of Medicine, University of Illinois at Chicago, Chicago, IL 60612, USA; htonc@uic.edu; 3 Department of Pharmacology, College of Medicine, University of Illinois at Chicago, Chicago, IL 60612, USA; smost@uic.edu; 4 Department of Kinesiology and Nutrition, College of Applied Health Sciences, University of Illinois at Chicago, Chicago, IL 60612, USA

**Keywords:** COVID-19, new-onset diabetes, post-acute sequelae (PASC), insulin resistance, pancreatic β-cell dysfunction, hyperglycemia, systemic inflammation, steroid-induced dysglycemia, long COVID, lifestyle interventions

## Abstract

Emerging evidence highlights the profound and lasting impact of severe illnesses such as COVID-19, particularly among individuals with underlying comorbidities. Patients with pre-existing conditions like diabetes mellitus (DM) are disproportionately affected, facing heightened risks of both disease exacerbation and the onset of new complications. Notably, the convergence of advanced age and DM has been consistently associated with poor COVID-19 outcomes. However, the long-term metabolic consequences of SARS-CoV-2 infection, especially its role in disrupting glucose homeostasis and potentially triggering or worsening DM, remain incompletely understood. This review synthesizes current clinical and experimental findings to clarify the bidirectional relationship between COVID-19 and diabetes. We critically examine literature reporting deterioration of glycemic control, onset of hyperglycemia in previously non-diabetic individuals, and worsening of metabolic parameters in diabetic patients after infection. Furthermore, we explore proposed mechanistic pathways, including pancreatic β-cell dysfunction, systemic inflammation, and immune-mediated damage, that may underpin the development or progression of DM in the post-COVID setting. Collectively, this work underscores the urgent need for continued research and clinical vigilance in managing metabolic health in COVID-19 survivors.

## 1. Introduction

COVID-19, caused by the severe acute respiratory syndrome coronavirus 2 (SARS-CoV-2), rapidly evolved into a global health crisis and was officially declared a pandemic by the World Health Organization (WHO) on 11 March 2020 [1]. The virus’s high transmissibility severely hindered efforts to control the spread, the severity of clinical manifestations, and the lag in developing effective vaccines, diagnostics, and treatments, further complicated by the continual emergence of new viral variants. Poor clinical outcomes have been most strongly associated with advanced age, comorbidities, and lack of vaccination. Notably, a bidirectional relationship has been observed between COVID-19 and certain metabolic disorders, particularly diabetes mellitus, though the precise mechanisms driving this interaction remain unclear. Unraveling this complex interplay is essential to inform the development of targeted therapeutic strategies and improve patient outcomes [2].

As the COVID-19 pandemic has progressed, substantial evidence has emerged indicating that many individuals experience persistent symptoms beyond the acute phase of infection. According to NIH definitions, post-acute sequelae of SARS-CoV-2 infection (PASC) refer to symptoms that persist for more than four weeks after the initial illness [3]. Estimates suggest that 20–30% of non-hospitalized and up to 50–89% of hospitalized patients develop these long-term complications. Common manifestations include fatigue, dyspnea, cognitive impairment (“brain fog”), and gastrointestinal disturbances. Importantly, these sequelae can impact both pulmonary and extrapulmonary systems, with growing concern surrounding their role in the onset or exacerbation of metabolic disorders, including type 1 and type 2 diabetes mellitus (T1DM and T2DM) [4].

Although T1DM accounts for only approximately 10% of all diabetes cases worldwide, its incidence is rising, particularly among younger populations. T1DM is primarily an autoimmune condition in which the immune system targets and destroys insulin-producing β-cells in the pancreas. A small subset of cases, classified as type 1B, lack evidence of autoimmunity, and their etiology remains unclear. The progression to insulin deficiency results from a complex interplay of genetic predisposition, environmental exposures, and immune dysregulation. This β-cell destruction typically occurs gradually over months or years in genetically susceptible individuals and is often precipitated by environmental triggers. During this preclinical phase, individuals are asymptomatic and maintain standard glycemic control, although diabetes-associated autoantibodies may be present. Clinical symptoms and overt hyperglycemia emerge only after a critical loss of β-cell function [5]. Notably, a growing number of studies have reported new-onset T1DM in individuals recovering from COVID-19, raising significant concerns about the virus’s potential role in precipitating autoimmune diabetes [6,7,8,9].

T2DM is primarily defined by chronic insulin resistance, in which peripheral tissues such as muscle, liver, and adipose fail to respond adequately to insulin. To compensate, pancreatic β-cells initially increase insulin production; however, this compensatory response becomes insufficient over time, leading to relative insulin deficiency and hyperglycemia. T2DM is a key component of metabolic syndrome, which encompasses a constellation of abnormalities, including central obesity, dyslipidemia, hypertension, and impaired glucose metabolism. While the exact pathogenesis is multifactorial, insulin resistance and β-cell dysfunction are central contributors, often driven by systemic inflammation, oxidative stress, lipotoxicity, and ectopic fat accumulation [10]. Recent studies have identified an increased incidence of T2DM in individuals recovering from COVID-19, suggesting that SARS-CoV-2 infection may act as a metabolic stressor capable of accelerating disease onset in predisposed individuals [11,12,13,14,15].

## 2. Incidence of Post-COVID-19 Diabetes

### 2.1. Epidemiological Evidence

Since the onset of the COVID-19 pandemic, individuals with diabetes mellitus (DM) have been identified as being at significantly higher risk for severe acute complications following SARS-CoV-2 infection. Moreover, emerging data indicate that these patients are also more susceptible to developing a broad spectrum of persistent symptoms collectively referred to as “Long COVID syndrome.” Increasing evidence further suggests that the clinical manifestations of long COVID may extend to include newly diagnosed DM [16], particularly during or shortly after COVID-19 infection, along with a notable increase in acute metabolic complications such as diabetic ketoacidosis (Table 1). Additionally, SARS-CoV-2 infection has been linked to elevated blood glucose levels even in individuals without a history of DM, underscoring its potential to disrupt glucose metabolism broadly [17].

A longitudinal study in Wenzhou, China, investigated β-cell function and insulin sensitivity in patients without pre-existing DM at baseline, 3 months, and 6 months post-hospitalization [18]. Using the Homeostasis Model Assessment of Insulin Resistance (HOMA-IR) and fasting C-peptide to assess β-cell activity and the triglyceride-glucose (TyG) index to estimate insulin resistance, the study found that while fasting blood glucose (FBG) initially declined post-discharge, it rose again at 6 months. Both TyG index and C-peptide increased significantly by 3 months and remained elevated, alongside lipid markers (cholesterol, triglycerides, LDL) and inflammatory indicators (CRP, uric acid, platelets, lymphocytes, hemoglobin). The observed hyperinsulinemia may reflect reduced insulin sensitivity in the peripheral metabolic tissues. Some limitations in this study include the lack of glucose tolerance testing, the absence of pre-infection metabolic data, and a geographically and ethnically limited sample. Nevertheless, the study proves that COVID-19 may induce insulin resistance even in individuals without prior DM [18].

A large-scale UK study assessed the post-discharge health outcomes of 47,780 individuals hospitalized with COVID-19, comparing them to a matched control group from the general population. Over a mean follow-up period of 140 days, patients who had been hospitalized with COVID-19 exhibited significantly higher rates of respiratory disease, new-onset DM, and cardiovascular complications [19]. In a separate retrospective cohort study involving 157,936 individuals aged ≥25 with confirmed COVID-19, researchers evaluated the risk of developing new-onset DM based on disease severity. Patients were stratified into non-hospitalized, hospitalized, and severely hospitalized groups and compared with matched controls. The analysis revealed no increased risk of DM among non-hospitalized individuals. In contrast, those who were hospitalized showed a significantly elevated risk, particularly during the acute phase (hazard ratio [HR] 2.47), with risk remaining elevated up to four months post-infection (HR 1.60) [20]. These findings underscore the severity-dependent impact of COVID-19 on long-term metabolic health and highlight the need for targeted follow-up in high-risk populations.

A comprehensive cohort study utilizing data from the U.S. Department of Veterans Affairs evaluated the risk of incident DM among individuals who survived the first 30 days following SARS-CoV-2 infection. The analysis included 181,280 COVID-19 patients, a contemporary control group of 4,118,441 individuals without documented infection during the same timeframe, and a historical control group of 4,286,911 individuals from the pre-pandemic era. Over a median follow-up of 352 days, COVID-19 survivors exhibited a significantly increased risk of developing new-onset DM (hazard ratio [HR] 1.40) and initiating antihyperglycemic therapy (HR 1.85) compared to the contemporary controls. These findings translated into excess burdens of 13.46 and 12.35 cases per 1000 persons at 12 months, respectively. Notably, the risk of DM increased proportionally with the severity of the acute infection, and results were consistent when benchmarked against the historical control group [21]. These data highlight the enduring metabolic consequences of COVID-19 and underscore the need for long-term monitoring of glycemic health in post-COVID populations.

Another retrospective cohort study leveraging data from the Veterans Health Administration investigated the association between SARS-CoV-2 infection and the risk of incident DM. The study analyzed 128,255 individuals with confirmed SARS-CoV-2 infection and 2,679,851 unexposed individuals without a documented positive test. Logistic regression analysis revealed a significant association between COVID-19 and increased DM risk in men, with odds ratios (OR) of 1.75 at 120 days and 1.44 throughout the follow-up period. In contrast, no significant association was observed in women. Among hospitalized patients, this elevated risk persisted in men but remained non-significant in women. These findings suggest a sex-specific vulnerability, indicating that SARS-CoV-2 infection is associated with a heightened risk of developing DM, particularly in men, independent of hospitalization-related surveillance bias [22].

Collectively, mounting evidence points to a significant association between SARS-CoV-2 infection and increased risk of new-onset DM. A recent meta-analysis encompassing over 5.7 million individuals revealed a 59% elevated risk of developing DM in the post-acute phase of COVID-19 compared to healthy controls (HR: 1.59; 95% CI: 1.40–1.81, *p* < 0.001) [23]. Notably, this risk remained higher compared to severity-matched non-COVID respiratory infections, with hospitalized COVID-19 cases showing a hazard ratio of 1.52 and mild cases with an HR of 1.22. Supporting these findings, a prospective cohort study in Puducherry, India, followed 724 individuals without prior DM and found that those with moderate to severe COVID-19 had a significantly increased risk of developing DM (RR: 2.83; adjusted RR: 2.01), with key contributing factors including older age, smoking, and pre-existing comorbidities [24]. While most large-scale studies classify new-onset diabetes as either type 1 or type 2, emerging case reports have described atypical presentations such as latent autoimmune diabetes in adults (LADA) following SARS-CoV-2 infection [25,26]. These findings highlight the need for vigilant long-term metabolic monitoring in COVID-19 survivors and underscore the urgency of defining distinct diabetes phenotypes that may emerge in the aftermath of viral infection. A summary of key studies investigating new-onset diabetes following COVID-19 recovery is provided in Table 1.

### 2.2. Divergent Diabetes Risks After COVID-19: Evidence for Type 1 and Type 2 Onset

A growing body of evidence has explored the relationship between SARS-CoV-2 infection and the development of both T1DM and T2DM. Several large-scale studies have specifically reported an increased incidence of T1DM following COVID-19. A cohort study involving nearly 2 million individuals found that recent SARS-CoV-2 infection (within the previous 30 days) was associated with a higher incidence of T1DM, particularly in children, whose rates rose by 20% during 2020–2021 compared to the prior seven-year average [8]. Similarly, a retrospective analysis of data from over 27 million patients revealed a significantly increased risk of new-onset T1DM post-COVID-19 diagnosis, with an overall odds ratio (OR) of 1.42. This risk varied across demographic groups and was notably higher in infants (0–1 years), American Indian/Alaskan Natives, Asian/Pacific Islanders, and adults aged 51–65. Additionally, among individuals with pre-existing T1DM, a COVID-19 diagnosis was associated with a significantly elevated risk of diabetic ketoacidosis (DKA) (OR 2.26), especially in those with higher comorbidity scores [9].

During a surge in pediatric T1DM cases in the spring of 2021, researchers found that 52.4% of newly diagnosed children (ages 0–18) had anti-SARS-CoV-2 spike antibodies, compared to only 22.7% of children with preexisting T1DM, yielding an OR of 3.74 [6]. Another large-scale analysis using electronic health records of over 1 million pediatric patients (≤18 years old) investigated the incidence of T1DM in children following COVID-19. After adjusting for demographics and family history, the study compared 285,628 children with COVID-19 to an equal number with non-COVID respiratory infections. Within six months, the incidence of new-onset T1DM was significantly higher in the COVID-19 group (0.043%) compared to the non-COVID group (0.025%). The risk remained significantly elevated at 1, 3, and 6 months post-infection, with hazard ratios of 1.96, 2.10, and 1.83, respectively. These trends were consistent across age groups, including younger children (0–9 years) and adolescents (10–18 years). The findings remained robust when using alternative comparison groups, such as children presenting with fractures or attending child wellness visits, reinforcing the potential association between COVID-19 and T1DM development [7].

In addition to evidence linking COVID-19 to an increased risk of T1DM, numerous studies have also demonstrated a heightened risk of developing T2DM following SARS-CoV-2 infection. Importantly, T2DM and COVID-19 share a bidirectional relationship: poorly controlled diabetes is associated with increased morbidity and mortality during acute COVID-19, while the infection itself, along with widespread use of corticosteroids during the pandemic, has contributed to the progression from prediabetes to overt T2DM, as well as the emergence of steroid-induced diabetes [27].

A large retrospective cohort study evaluated the long-term health outcomes of 193,113 adults aged 18–65 who had COVID-19, comparing them to three control groups: individuals without COVID-19 in 2020, a 2019 historical cohort, and patients with non-COVID viral lower respiratory tract illness. Within this group, 14% (27,074 individuals) developed at least one new health condition requiring medical attention after the acute phase, representing a 4.95% increase compared to the 2020 control group. COVID-19 was significantly associated with an increased risk of more than 50 health complications, including new-onset diabetes, respiratory failure, cardiac arrhythmias, hypercoagulability, encephalopathy, neuropathy, cognitive impairment, liver dysfunction, myocarditis, anxiety, and fatigue [14]. Similarly, compared to patients with pneumonia, those with COVID-19 had a significantly higher risk of developing T2DM (subdistribution hazard ratio [SHR] 1.46) [13].

Further supporting this link, a study involving 70 million patients compared 600,055 individuals diagnosed with COVID-19 to 394,667 with influenza. The risk of developing T2DM within 180 days was 1.54 times higher in those with mild COVID-19 and 1.46 times higher in those with moderate or severe disease. After excluding individuals who had received corticosteroids, the relative risk (RR) for mild cases decreased slightly to 1.22, while the risk remained unchanged for more severe cases. Notably, the incidence rate of T2DM was substantially higher in moderate/severe COVID-19 (83 per 1000 person-years) compared to mild cases (23 per 1000 person-years), suggesting that the severity of infection plays a critical role in metabolic outcomes [11].

**Figure 1:**
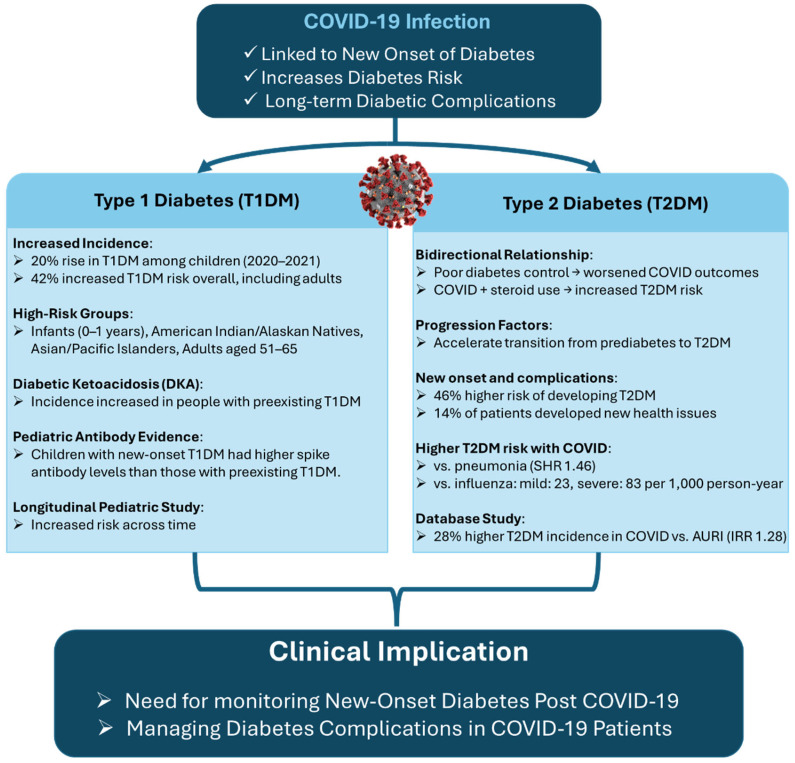
Divergent diabetes risks following COVID-19 infection. This figure compares the impact of COVID-19 on the incidence and progression of type 1 (T1DM) and type 2 diabetes mellitus (T2DM). T1DM findings highlight increased incidence in children, elevated DKA risk, and autoimmune features post-infection. T2DM is associated with a bidirectional relationship with COVID-19, higher incidence following infection, especially with steroid use, and greater risk than pneumonia and influenza. The diagram emphasizes the need for post-COVID diabetes screening and long-term management.

Another retrospective analysis using a database of 8.8 million patients compared 35,865 individuals with COVID-19 to a propensity-matched cohort with acute upper respiratory tract infections (AURI). After adjusting for age, sex, and comorbidities and excluding those on corticosteroids, the study found a statistically significant 28% increase in the incidence rate of T2DM (IRR 1.28) in the COVID-19 group. These findings underscore the importance of monitoring glucose levels even after mild SARS-CoV-2 infections and suggest that COVID-19 may precipitate T2DM in at-risk individuals [15]. A visual summary of these divergent risks and outcomes for T1DM and T2DM following COVID-19 infection is presented in Figure 1.

## 3. Iological Underpinnings of COVID-19–Related Diabetes

### 3.1. Direct Viral Impact on Pancreatic Beta Cells

Growing evidence suggests that SARS-CoV-2 infection can directly contribute to islet cell injury and the development of T1DM [28]. The severity of COVID-19 has been shown to correlate with the risk of developing diabetes, with mild disease associated with an incidence of 23 per 1000 person-years and moderate-to-severe disease with a significantly higher rate of 83 per 1000 person-years [11]. This dose-response relationship, observed across multiple cohorts [21], implies that greater viral load during acute infection may elevate the risk of new-onset T1DM. Comparative studies indicate that SARS-CoV-2 presents a higher diabetogenic risk than influenza [11]. A key mechanism underlying the pathophysiology of SARS-CoV-2 involves its entry into host cells via the angiotensin-converting enzyme 2 (ACE2) receptor. ACE2 has been identified as a critical mediator of viral invasion and disease progression, with high expression levels in the lungs and other vital organs making these tissues particularly susceptible to infection and damage [29]. Notably, previous studies on SARS-CoV, closely related to SARS-CoV-2, have shown that infection can lead to new-onset DM that persists for years, likely through a similar ACE2-dependent mechanism [30].

SARS-CoV-2 gains cellular entry by binding its spike glycoproteins to the ACE2 receptor. This process reduces ACE2 availability, allowing unopposed angiotensin II activity, which promotes aldosterone release and triggers pro-inflammatory signaling via angiotensin II receptors AT1R and AT2R [31]. These downstream effects contribute to systemic inflammation and tissue injury. Importantly, therapeutic agents such as angiotensin receptor blockers (ARBs) and ACE inhibitors (ACEIs), which upregulate ACE2 expression, have been hypothesized to potentially increase susceptibility to SARS-CoV-2 by enhancing viral entry into host cells, thereby raising concerns about their use during active infection [31]. Supporting this mechanism, a detailed analysis of pancreatic autopsy tissues from deceased COVID-19 patients, employing immunohistochemistry and electron microscopy, confirmed SARS-CoV-2 infiltration of pancreatic β-cells. Experiments using SARS-CoV-2 pseudoviruses demonstrated that isolated human islet cells are indeed permissive to infection [32]. While ACE2 was detected in 70% of vascular tissues, only 30% of β-cells exhibited ACE2 expression, suggesting alternative entry pathways. The study identified additional host factors that may facilitate viral entry into islet cells, including TMPRSS2, DPP4, HMGB1, and neuropilin-1 (NRP1), highlighting a multifactorial route by which SARS-CoV-2 may access and damage the endocrine pancreas [32].

SARS-CoV-2 has been shown to directly target the endocrine pancreas, infecting and replicating within pancreatic β-cells. This viral invasion leads to substantial β-cell damage, characterized by reduced insulin-secreting granules, impaired proinsulin and insulin secretion, and β-cell transdifferentiation or dedifferentiation [33,34]. These disruptions result in decreased insulin output and compensatory increases in glucagon and trypsin-1 secretion, further contributing to glycometabolic imbalance [35]. In addition to direct cytopathic effects, SARS-CoV-2 manipulates host cellular signaling to facilitate viral replication. Viral proteins can upregulate or suppress the activity of nearly 100 human kinases involved in cellular metabolism, immune regulation, and signal transduction [36]. This includes downregulating key pathways integral to insulin function, specifically the insulin signaling, mTOR, and MAPK pathways [37].

Significant disruptions have been observed in critical insulin/IGF signaling components such as insulin receptor substrate (IRS), PI3K, AKT, and mTOR, proteins essential for maintaining glucose homeostasis, energy metabolism, and cell viability. Disruption of the insulin/IGF signaling pathway during SARS-CoV-2 infection has been mechanistically linked to heightened interferon responses, particularly through the upregulation of interferon regulatory factor-1 (IRF-1) [37,38]. IRF-1 is a transcriptional modulator capable of modulating gene expression to finely regulate the immune response. SARS-CoV-2 infection significantly increases IRF-1 expression, which in turn impairs the insulin/IGF signaling cascade, critical for maintaining metabolic balance [37]. Dysfunction of this pathway in insulin-sensitive organs contributes to systemic insulin resistance, metabolic dysregulation, and cellular apoptosis. In the pancreas, SARS-CoV-2 directly infects β-cells, inducing necroptotic cell death and severely compromising insulin production [32]. Viral infection leads to a marked reduction in pancreatic insulin levels and secretory capacity, alongside β-cell apoptosis. Notably, these effects are mitigated by inhibition of NRP1, a co-receptor that facilitates viral entry into islet cells [39].

**Figure 2:**
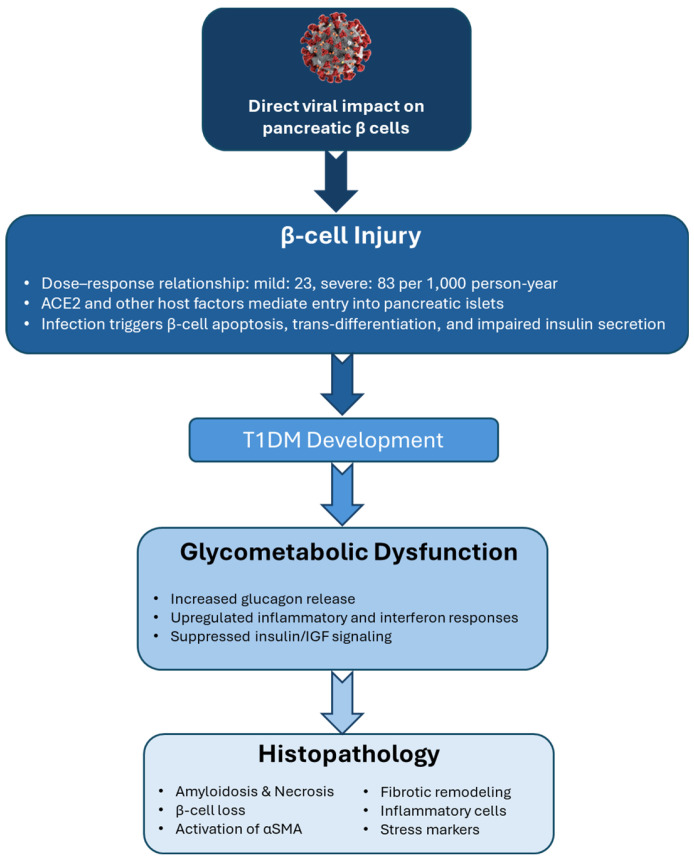
Direct viral injury to pancreatic β-cells and the pathogenesis of T1DM. This diagram illustrates how SARS-CoV-2 may contribute to type 1 diabetes (T1DM) development through direct β-cell infection. Viral entry via ACE2 and other host factors leads to β-cell injury, glycometabolic dysfunction, and histopathological changes, including cell loss and inflammation, ultimately promoting T1DM onset.

Phosphoproteomic profiling of infected islets reveals activation of apoptotic signaling pathways consistent with those observed in T1DM, reinforcing the hypothesis that SARS-CoV-2 can directly induce β-cell destruction [39]. This is further supported by autopsy findings from 67 non-human primates infected with SARS-CoV-2, which showed significant loss of β-cells and diminished insulin expression in situ. Histopathological features included islet amyloidosis, necrosis, α-smooth muscle actin (αSMA) activation, and fibrotic remodeling characterized by reduced serum collagen levels. These structural alterations were accompanied by elevated expression of inflammatory and stress markers, including ICAM-1 and G3BP1, and were associated with severe glycometabolic dysfunction leading to islet cell death [40]. Figure 2 illustrates the proposed pathway by which direct viral injury to pancreatic β-cells contributes to the development of T1DM and downstream glycometabolic dysfunction.

### 3.2. Immune-Mediated Mechanisms

Emerging evidence suggests that SARS-CoV-2 may contribute to diabetes onset or progression through immune-mediated pathways, including initiating or accelerating autoimmune responses and heightened systemic inflammation. A study from Massachusetts General Hospital led by Cromer and colleagues [41] analyzed 1902 patients hospitalized with COVID-19, of whom 594 (31.2%) had DM. Notably, 77 patients (13.0% of the DM group) were newly diagnosed with diabetes during their admission. Compared to those with pre-existing diabetes, newly diagnosed patients were generally younger and exhibited lower glycemic indices and insulin requirements but experienced more extended hospital stays, elevated inflammatory markers, and a higher likelihood of requiring intensive care, though not an increased risk of mortality. Among the 64 newly diagnosed patients who survived, 36 (56.3%) maintained a diabetes diagnosis during follow-up, while 26 (40.6%) showed regression to normoglycemia or prediabetes. In a separate pediatric study, researchers explored the potential link between SARS-CoV-2 infection and increased incidence of T1DM in children. In this study, all new T1DM cases tested positive for at least one diabetes-related autoantibody, suggesting that SARS-CoV-2 may accelerate an existing autoimmune process or unmask latent T1DM [6].

A marked increase in immune cell infiltration has been observed in the pancreatic islet tissue of COVID-19 patients compared to non-infected controls [42]. While both endocrine and exocrine compartments of the pancreas exhibit lymphocytic infiltration, the islet cells appear preferentially affected in some instances, suggesting a higher β-cell-specific viral load in these individuals. Lymphocyte presence has also been noted in the peripancreatic tissue, reinforcing the hypothesis that SARS-CoV-2 may contribute to β-cell dysfunction through direct and indirect mechanisms. The virus is known to induce local pancreatic inflammation via direct infection of β-cells through ACE2 receptor binding and broader systemic inflammatory responses [32]. Another proposed mechanism for post-COVID T1DM is molecular mimicry. This process occurs when viral epitopes share structural similarities with host islet antigens, potentially provoking an autoimmune attack on β-cells in genetically predisposed individuals. Rather than initiating autoimmunity de novo, molecular mimicry may act as an accelerator of an already primed autoimmune process, hastening the clinical onset of T1DM [43].

Prolonged SARS-CoV-2 infection of pancreatic β-cells can lead to sustained overexpression of major histocompatibility complex class I (MHC-I) molecules. This persistent MHC-I upregulation promotes the continuous presentation of β-cell-specific epitopes to the immune system, thereby facilitating the development and perpetuation of autoimmunity [44]. As β-cells are progressively destroyed, additional islet antigens are exposed, a process known as epitope spreading, which amplifies the autoimmune response. This leads to the activation of cytotoxic CD8+ T cells and the production of a wider array of islet-directed autoantibodies targeting antigens such as insulin, glutamic acid decarboxylase (GAD), and protein tyrosine phosphatase (IA-2). The resulting immune-mediated depletion of functional β-cells culminates in hyperglycemia and the clinical onset of T1DM [45]. Moreover, the exaggerated formation of neutrophil extracellular traps (NETs) during COVID-19 infection may further exacerbate autoimmune processes. NETs are web-like structures composed of DNA, histones, and antimicrobial enzymes released by neutrophils to trap and neutralize pathogens. While NETs play a protective role in host defense, their dysregulated and excessive formation can cause autoimmune inflammation and collateral tissue damage, potentially contributing to β-cell autoimmunity and islet injury [46].

ACE2 receptor exerts anti-inflammatory and protective effects by converting angiotensin II into angiotensin 1–7, a peptide with vasodilatory, antifibrotic, and anti-inflammatory properties. SARS-CoV-2 infection leads to a downregulation of ACE2 expression, reducing angiotensin 1–7 availability and consequently amplifying inflammation, oxidative stress, and coagulability [47]. The SARS-CoV-2 spike (S) protein facilitates viral entry by binding to the ACE2 receptor on host cells, initiating intracellular replication and tissue damage [48]. Upon entry, SARS-CoV-2 activates host pattern recognition receptors, particularly Toll-like receptors (TLRs), which trigger a robust innate immune response. This activation leads to the release of a cascade of proinflammatory cytokines, including tumor necrosis factor-alpha (TNF-α), interleukins (IL-2, IL-4, IL-6, IL-7), interferon-gamma (IFN-γ), and nuclear factor kappa B (NF-κB) [21,36]. These cytokines recruit additional immune cells, such as macrophages and monocytes, and prime the adaptive immune response by activating T and B lymphocytes for targeted defense [49]. However, in some cases, this immune activation becomes dysregulated, culminating in a hyperinflammatory state known as a “cytokine storm” [48].This pathological amplification of inflammation can lead to widespread tissue damage and multi-organ dysfunction, including injury to the pancreas, the primary site of insulin production [50,51]. The resulting inflammatory milieu may impair pancreatic β-cell function, further contributing to the development or exacerbation of diabetes in COVID-19 patients [52].

Chen et al. reported that patients who died due to COVID-19 exhibited significantly elevated levels of inflammatory cytokines, including IL-2 receptors, IL-10, TNF-α, IL-6, and IL-8, compared to those who recovered [18]. Similarly, a study by Queiroz et al. [53] identified a distinct cytokine profile in individuals with long COVID, characterized by elevated IL-17 and IL-2 and reduced levels of IL-4 and IL-10, suggesting a persistent proinflammatory immune state [54]. Extensive research has demonstrated that pro-inflammatory cytokines involved in the acute-phase response, such as IL-6, TNF-α, and interferons, play a significant role in developing T2DM by promoting insulin resistance and metabolic disruption [51,55,56]. Both in vitro and in vivo studies have consistently demonstrated that elevated TNF-α levels are associated with reduced insulin sensitivity, particularly in skeletal muscle and adipose tissue, two key insulin-responsive compartments [57,58]. Even modest elevations in TNF-α can impair peripheral insulin action by downregulating GLUT4, a key glucose transporter in insulin-sensitive tissues, thereby reducing glucose uptake and storage in skeletal muscle [57,59]. To mitigate the inflammatory response associated with COVID-19, tocilizumab, a humanized monoclonal antibody targeting the IL-6 receptor, has been employed as an adjuvant therapy. Among hospitalized patients with new-onset hyperglycemia, those treated with tocilizumab demonstrated significantly greater reductions in blood glucose levels at discharge compared to those who did not receive the drug, highlighting its potential dual benefit in controlling both inflammation and glycemic dysregulation [60].

**Figure 3:**
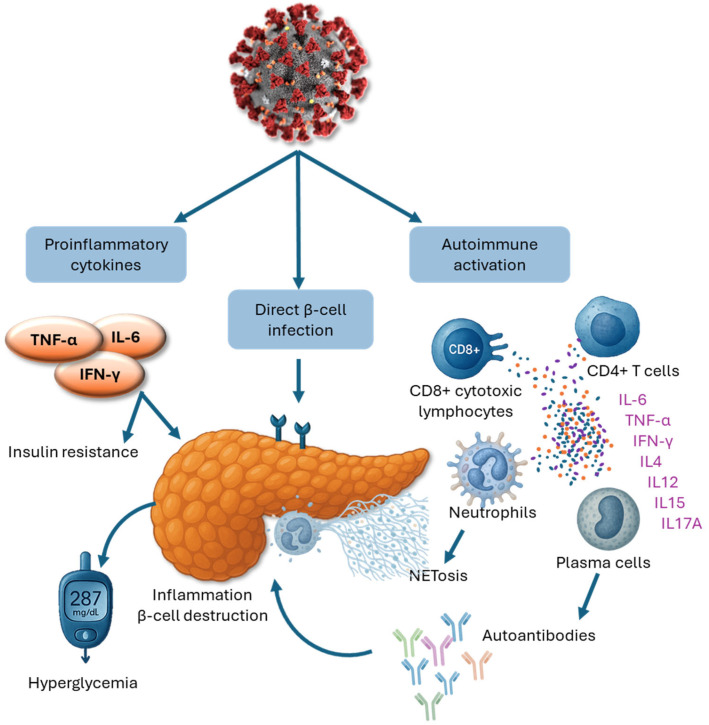
Immune-mediated and direct viral mechanisms of pancreatic β-cell damage in COVID-19. This diagram illustrates the proposed pathways by which SARS-CoV-2 infection may contribute to β-cell injury and diabetes development. Viral entry into β-cells via ACE2 receptors leads to direct cytotoxic effects, while elevated levels of proinflammatory cytokines (TNF-α, IL-6, IFN-γ) promote insulin resistance and hyperglycemia. Concurrently, autoimmune activation results in CD8+ T cell infiltration, cytokine release, and neutrophil extracellular trap (NET) formation. These immune responses, along with the generation of islet autoantibodies, contribute to progressive β-cell dysfunction and diabetes onset.

The relationship between DM and COVID-19 is bidirectional: while SARS-CoV-2 infection may precipitate new-onset diabetes or worsen glycemic control, pre-existing or uncontrolled diabetes can also adversely impact COVID-19 outcomes. Uncontrolled hyperglycemia not only impairs host immune function but may also exacerbate COVID-19 severity by enhancing viral spike protein binding to the ACE2 receptor and amplifying the immune response [28]. During critical stages of infection, the interplay between viral replication and immune activation can further elevate glucose levels and reduce insulin sensitivity, compounding metabolic complications [61]. Moreover, SARS-CoV-2 infection can stimulate the release of stress hormones such as adrenaline and cortisol, which promote gluconeogenesis and contribute to hyperglycemia [62]. Prolonged hyperglycemia, in turn, impairs both innate and adaptive immunity by inhibiting lymphocyte proliferation, reducing natural killer cell activity, and compromising the function of monocytes, macrophages, and neutrophils [63]. Elevated blood glucose levels at the time of hospital admission have been independently associated with a greater risk of critical disease progression and mortality in COVID-19 patients [64]. Furthermore, patients with hyperglycemia during hospitalization exhibit higher levels of IL-6 and D-dimer, markers indicative of systemic inflammation and hypercoagulability. Notably, achieving effective glucose control significantly reduces these biomarkers, suggesting that hyperglycemia exacerbates inflammatory and thrombotic responses independently of viral factors [65]. Figure 3 summarizes the combined effects of direct viral invasion and immune-mediated pathways by which SARS-CoV-2 may promote pancreatic β-cell damage and autoimmunity, leading to diabetes.

### 3.3. Impact of COVID-19 Therapies on Glycemic Control and Diabetes Risk

Several pharmacological agents commonly used to treat COVID-19, including corticosteroids, antiviral drugs, and glucose-containing antitussive syrups, are known to elevate blood glucose levels, thereby increasing the risk of metabolic complications. Corticosteroids such as dexamethasone, as well as antiviral agents like remdesivir, lopinavir, and ritonavir, have all been shown to impair glycemic control significantly. Vaccines, including Covishield, Pfizer-BioNTech, and Moderna, have also been linked to transient hyperglycemia following the first dose [61,66]. While these therapeutic strategies are essential for the immediate management of acute and severe COVID-19, they can inadvertently exacerbate pre-existing metabolic disorders such as diabetes. In particular, systemic corticosteroids, routinely administered to suppress the hyperinflammatory response in critically ill patients, play a central role in COVID-19 treatment protocols. Although effective in reducing the need for mechanical ventilation and lowering all-cause mortality, corticosteroids, especially at high doses, can lead to hyperglycemia, hypertension, and increased susceptibility to secondary infections. Thus, the therapeutic benefits of these agents must be carefully weighed against their potential to contribute to post-COVID glycemic dysregulation, particularly in vulnerable populations.

Corticosteroids can be delivered orally or via injection, offering broad systemic effects. However, their short-term use, especially at high doses, has been associated with adverse outcomes, including elevated blood glucose and blood pressure, as well as increased susceptibility to secondary infections, such as opportunistic fungal infections. Patients with more severe COVID-19 often receive more aggressive corticosteroid regimens to manage acute symptoms. However, this therapeutic approach, while addressing immediate clinical needs, may inadvertently heighten the risk of post-COVID hyperglycemia and contribute to the development or exacerbation of diabetes.

### 3.4. Post-COVID Insulin Resistance: A Persistent Metabolic Consequence

Insulin resistance, the diminished cellular response to insulin, is a defining feature of metabolic disorders such as T2DM. It is commonly accompanied by compensatory hyperinsulinemia, where the pancreas increases insulin production to maintain normal glucose levels. In a study assessing insulin resistance and β-cell function in hospitalized COVID-19 patients, researchers performed fasting serum hormone sampling following arginine stimulation [60]. Compared to uninfected controls, hospitalized COVID-19 patients exhibited significantly elevated fasting insulin levels, C-peptide, and HOMA-IR. Notably, these metabolic disturbances persisted beyond the acute phase. Patients assessed two months after disease onset continued to show higher fasting insulin, C-peptide, and HOMA-IR levels than healthy controls. Additionally, the area under the curve (AUC) for fasting insulin was increased in the recovery group, whereas C-peptide levels did not show a corresponding rise. Since insulin and C-peptide are typically secreted in equimolar amounts, this discrepancy may reflect impaired insulin clearance or an imbalance between insulin secretion and degradation during recovery. These findings indicate that even after clinical recovery, COVID-19 survivors without a prior history of diabetes can exhibit ongoing metabolic dysfunction suggestive of persistent insulin resistance. The elevated HOMA-IR, insulin, and C-peptide levels point to a state where higher insulin concentrations are required to maintain euglycemia, consistent with mechanisms observed in T2DM. Moreover, the combination of elevated insulin secretion and possible clearance defects highlights a complex disruption in insulin homeostasis following SARS-CoV-2 infection. Collectively, the data suggest that COVID-19 may induce long-term metabolic alterations comparable to those seen in type 2 diabetes.

Patients with COVID-19 frequently develop reduced insulin sensitivity due to SARS-CoV-2–induced β-cell damage, often necessitating increased insulin dosages, particularly during febrile episodes when metabolic demand is heightened [59]. Compelling evidence from a study by He et al. [67] demonstrated that COVID-19 can induce new-onset insulin resistance even in individuals without any prior history of metabolic disease. The study identified a downregulation in the activity of the REST transcription factor, which plays a key role in regulating genes critical to glucose and lipid metabolism. Furthermore, elevated levels of short-chain fatty acids, specifically propionic and isobutyric acids, were observed, which have been previously implicated in developing insulin resistance in animal models. Notably, the participants in this study had BMI values ranging from 20.5 to 24.6, indicating that even lean individuals, typically considered low risk, may be susceptible to post-COVID metabolic dysfunction. Notably, the findings also revealed that insulin resistance persisted even after the virus was cleared from the body, highlighting the potential for long-term metabolic consequences in COVID-19 survivors. These results suggest that SARS-CoV-2 may trigger durable alterations in metabolic regulation, independent of conventional risk factors such as obesity or pre-existing diabetes [67].

Acute inflammation during SARS-CoV-2 infection induces significant cellular stress, which can accelerate lipolysis and increase circulating free fatty acids, contributing to relative insulin deficiency and impaired glucose homeostasis [68]. This stress also activates the integrated stress response (ISR), a protective cellular mechanism that engages a network of four serine/threonine kinases. Among these are RNA-dependent protein kinases capable of phosphorylating insulin receptor substrates (IRS) at serine residues, thereby disrupting the insulin signaling cascade and promoting insulin resistance. SARS-CoV-2 infection further exacerbates this process. Viral RNA fragments can directly activate these kinases, intensifying the ISR and impairing insulin action at the molecular level [47]. In addition, the cytokine storm frequently observed in severe COVID-19 cases also stimulates this kinase family, suppressing insulin signaling and deepening insulin resistance [69,70]. Moreover, viral infection-induced production of IFN-γ has been shown to downregulate insulin receptor expression in skeletal muscle, further compromising insulin sensitivity. This mechanism is particularly relevant to insulin resistance in adult and pediatric patients with COVID-19-associated diabetes [71,72,73].

### 3.5. SARS-CoV-2 and Systemic Metabolic Injury

ACE2 receptors are widely expressed throughout the human body, including in metabolically active tissues such as adipose tissue, the liver, and the small intestine [74]. ACE2 plays a critical role in modulating glucose absorption in the intestinal epithelium by regulating the sodium-glucose co-transporter 1 (SGLT1) in enterocytes. Upon binding the SARS-CoV-2 spike protein, ACE2 is internalized and downregulated, leading to compensatory upregulation of SGLT1. This dysregulation promotes excessive glucose absorption, contributing to hyperglycemia. 

Beyond its role in the gut, ACE2 also plays a critical metabolic function in the liver, where its disruption by SARS-CoV-2 contributes to glucose dysregulation through distinct hepatic mechanisms. Hepatocytes express both ACE2 receptors and transmembrane serine proteases, which are essential for SARS-CoV-2 entry into cells [75]. Once inside, the virus promotes hepatic glucose production by activating gluconeogenic pathways. Molecular analyses have shown that SARS-CoV-2 upregulates key enzymes involved in gluconeogenesis, particularly phosphoenolpyruvate carboxykinase (PEPCK), a rate-limiting enzyme in this pathway [76]. Biochemically, SARS-CoV-2 appears to shift cellular metabolism toward hyperglycolysis, likely to sustain lactate production, which, along with other gluconeogenic substrates such as alanine and glutamate, fuels hepatic glucose output. This shift ensures a continuous supply of gluconeogenic precursors and may drive excessive glucose production during infection. Moreover, hypoxic conditions resulting from impaired ventilation or septic shock caused by cytokine storms can further enhance glycolytic activity, compounding hyperglycemia in COVID-19 patients. In addition, experimental studies have shown that direct exposure of insulin-sensitive cells to lactate induces insulin resistance, suggesting that the metabolic reprogramming induced by SARS-CoV-2 may play a significant role in the onset or exacerbation of T2DM [77].

Liver injury has emerged as a frequent and clinically significant complication in patients with COVID-19, particularly in those with severe or critical illness. Manifestations of direct hepatic damage include elevated aminotransferases (ALT and AST), increased bilirubin levels, and reduced albumin, the most abundant plasma protein, reflecting impaired synthetic liver function. Elevated AST and bilirubin levels correlate strongly with ICU admission and disease severity, while persistently elevated ALT levels post-recovery are associated with an increased risk of rehospitalization and mortality within 30 days. Clinical data show that up to 43.4% of COVID-19 patients experience some degree of hepatic abnormality, with liver injury present in 74.4% of those with severe infection, compared to significantly lower rates in milder cases. Moreover, liver damage was reported in 58% of patients who succumbed to the disease [78]. Autopsy findings support these observations, revealing SARS-CoV-2 viral particles not only in the pulmonary and parenchymal tissues but also in the vascular endothelium of the liver. RT-PCR has also confirmed the presence of viral RNA in hepatic tissue, implicating direct viral invasion, vascular injury, and immune-mediated inflammation as potential contributors to hepatocellular damage. Finally, drug-induced hepatotoxicity further compounds liver vulnerability. For example, ribavirin, an antiviral often used against RNA viruses, has been linked to significant hepatotoxicity and hemolytic effects following discontinuation. Corticosteroids, widely used for their anti-inflammatory properties, may also exacerbate liver injury, particularly with prolonged or high-dose use [78].

Given the high prevalence and clinical consequences of hepatic involvement, there is a critical need to prioritize the study and monitoring of liver function in COVID-19 patients, both during acute management and long after recovery. Understanding and mitigating hepatic complications will be essential to reducing long-term metabolic sequelae associated with SARS-CoV-2 infection. This multifactorial injury compromises hepatic glucose metabolism, further contributing to hyperglycemia during and after infection. Adipose tissue has also emerged as a critical metabolic target of SARS-CoV-2, with growing evidence implicating it in the pathogenesis of insulin resistance and post-COVID hyperglycemia. Direct infection of adipose tissue by SARS-CoV-2 has emerged as a potential mechanism underlying adipose tissue dysfunction and insulin resistance in COVID-19. Adipose dysfunction is increasingly recognized as a metabolic hallmark of COVID-19 that may contribute to hyperglycemia. In animal models, SARS-CoV-2 viral RNA has been detected in adipose tissue of infected hamsters, accompanied by significant downregulation of Adipoq, the gene encoding adiponectin, a key insulin-sensitizing adipokine [79]. This is associated with a shift toward a proinflammatory, antiviral secretory phenotype in host adipocytes [47]. In clinical studies, both adiponectin levels and adiponectin-to-leptin ratios, widely used as insulin sensitivity indicators in T2DM, are significantly reduced in COVID-19 patients [79,80,81].

Furthermore, SARS-CoV-2 has been shown to infect and replicate across various adipose depots, triggering a robust inflammatory response involving two key cellular targets: adipocytes and a subset of inflammatory adipose tissue-resident macrophages. These findings suggest that adipose tissue is not only permissive to viral replication but may also act as a reservoir for prolonged viral persistence. The sustained presence of SARS-CoV-2 in adipose tissue likely contributes to ongoing local and systemic inflammation, exacerbating insulin resistance and potentially driving the onset of new diabetes in previously non-diabetic individuals [82].

SARS-CoV-2 also targets the vascular system, where it induces endothelial injury that further disrupts metabolic homeostasis and contributes to glycemic dysregulation [83]. This vascular insult is further amplified by the excessive release of pro-inflammatory cytokines during COVID-19, which exacerbates endothelial dysfunction and contributes to systemic hyperglycemia [84]. Compounding this effect, the downregulation of ACE2 and the unopposed action of angiotensin II result in impaired microvascular perfusion, including reduced blood flow to pancreatic β-cells. This ischemic stress contributes to β-cell dysfunction and subsequent glucose dysregulation, further linking vascular impairment to the pathogenesis of COVID-19-associated glycemic dysregulation [52].

Similarly, the kidneys are highly susceptible to injury during COVID-19 infection, especially given their essential roles in regulating blood volume, pressure, electrolyte balance, acid-base homeostasis, and the clearance of toxins, free radicals, and metabolic waste. Impairment of renal function under these conditions can have life-threatening consequences. Patients with pre-existing chronic kidney disease, often secondary to DM, face an elevated risk of developing acute kidney injury (AKI) when hospitalized with severe or critical COVID-19. One key mechanism underlying COVID-19-related AKI is renal hypoperfusion, which can result from systemic hypotension and reduced cardiac output. Critically ill patients who require deep sedation, mechanical ventilation, and high positive end-expiratory pressure (PEEP) are particularly vulnerable to these hemodynamic disturbances [85].

To further investigate renal complications, a study employed multiparametric magnetic resonance imaging (MRI) to assess renal perfusion, oxygenation, and water diffusion in ICU patients with COVID-19, both with and without AKI. The cohort included 19 patients aged 61–72 with acute respiratory failure, compared to healthy controls aged 58–73. Many had comorbidities such as DM and hypertension and were treated with ACE inhibitors or angiotensin receptor blockers before admission. Dexamethasone was administered as part of clinical care. Findings revealed that total cortical and medullary blood flow was significantly reduced in patients with AKI compared to those without. Interestingly, despite this marked reduction in renal perfusion, renal oxygenation remained largely unchanged, suggesting hypoperfusion, rather than hypoxia, is an early hallmark of AKI in COVID-19. These results underscore the unique pathophysiological profile of COVID-associated renal injury and the importance of closely monitoring kidney function in critically ill patients, particularly those with diabetes or other underlying vulnerabilities [85].

## 4. Demographic Disparities in Post-COVID Diabetes Risk

Understanding the demographic patterns associated with post-COVID diabetes is critical for identifying at-risk populations and addressing emerging health disparities. A study by Qeadan et al. [9] evaluated the risk of new-onset T1DM following COVID-19 infection in a retrospective study that analyzed data from 27,292,879 patients using the Cerner Real-World Data platform. Findings from this study revealed a significant association between COVID-19 diagnosis and increased risk of subsequent T1DM. Certain demographic groups were disproportionately affected, with the highest odds observed among pediatric patients aged 0–1 year (OR: 6.84; 95% CI: 2.75–17.02). Elevated risks were also reported in American Indian/Alaskan Native (OR: 2.30; 95% CI: 1.86–2.82), Asian/Pacific Islander (OR: 2.01; 95% CI: 1.61–2.53), and Black populations (OR: 1.59; 95% CI: 1.47–1.71), as well as in older adults aged 51–65 years (OR: 1.77; 95% CI: 1.66–1.88). Individuals residing in the northeast (OR: 1.71; 95% CI: 1.61–1.81) and the west US geographical regions (OR: 1.65; 95% CI: 1.56–1.74) also demonstrated elevated risk. These results highlight the potential impact of COVID-19 on triggering autoimmune diabetes and underscore disparities in vulnerability across age, racial, and geographic groups.

A study by Wander et al. [22] offered additional evidence that the risk of new-onset diabetes following SARS-CoV-2 infection is influenced by sex, race, and age. This study reported that COVID-19 was associated with a significantly higher risk of incident diabetes in men but not in women, even after adjusting for potential surveillance bias related to hospitalization. This finding is consistent with results from Sylvester et al. [86], which showed that males were significantly more likely to develop post-COVID diabetes (OR = 0.75; 95% CI: 0.69–0.81). However, other studies have reported no significant sex-based differences in diabetes incidence following COVID-19 infection [4,19,21]. Racial and ethnic disparities have also been documented. The incidence of post-COVID diabetes was higher among Black, Latinx, and other minority ethnic groups compared to white populations [22]. These disparities likely reflect longstanding structural inequities, including higher rates of pre-existing comorbidities (e.g., diabetes), increased exposure risk (e.g., overcrowded housing, essential worker status), and limited access to healthcare resources (e.g., insurance coverage and tertiary care facilities) [87]. Age also appears to be a contributing factor. Several studies, including that of Jayaseelan et al. [24], have identified individuals over the age of 40 as being at significantly higher risk for developing diabetes after COVID-19. While many reports support the link between older age and increased post-COVID diabetes risk [19,21], a few studies have suggested that age may not be a consistent predictor [14,88].

## 5. Targeting Post-COVID-19 Diabetes Through Lifestyle Modification

Emerging evidence highlights the critical role of lifestyle factors in preventing severe acute outcomes, mitigating the risk of lingering post-COVID sequelae, and accelerating metabolic recovery. Supporting this connection, a large population-based study demonstrated that individuals who adhered to a healthy lifestyle prior to contracting COVID-19 experienced significantly lower risks of developing multisystem complications, hospitalization, and death across various organ systems and symptoms, regardless of vaccination status, viral variant, or disease severity [89]. Notably, the protective effect of a healthy lifestyle persisted after adjusting for pre-existing conditions and exceeded the benefits of some pharmaceutical interventions. These findings underscore the limitations of current therapies and vaccines in preventing long COVID and highlight the pivotal role of modifiable lifestyle factors, such as BMI, sleep duration, and sedentary behavior, in shaping post-COVID outcomes. In parallel, well-established risk behaviors such as active and passive cigarette smoking markedly increase the incidence of diabetes and its complications. Reducing or eliminating tobacco exposure has been shown to lower the risk of both diabetes and cardiovascular disease. Similarly, excessive alcohol consumption is a known contributor to the development of diabetes, particularly T2DM. Among young individuals with T1DM, alcohol use presents an additional challenge due to its disruptive effects on blood glucose regulation, posing serious short- and long-term health risks [90].

These insights are further supported by behavioral trends observed during the pandemic. While some individuals reported improvements in sleep duration, there was a concurrent rise in sedentary behaviors and weight gain and a decline in physical activity, all of which are key contributors to poor metabolic outcomes [91,92]. These changes underscore the importance of habit reform, particularly during times of social disruption, as a means to enhance the effectiveness of diabetes care and long-term disease management. Moreover, even short-term periods of physical inactivity, increased sedentary behavior, and poor dietary choices have been shown to elevate insulin resistance, increase total and abdominal fat mass, raise levels of inflammatory cytokines, and impair endothelial function. These changes collectively contribute to heightened cardiometabolic risk, immunosenescence, and worsened blood pressure and heart rate profiles, all of which complicate diabetes management and recovery from COVID-19 [93]. Taken together, the risk factors for post-COVID diabetes closely mirror those associated with T2DM, reinforcing the hypothesis that COVID-19 may act as an accelerant for diabetes in individuals already predisposed due to conditions like obesity or prediabetes [94]. This highlights the urgent need to prioritize lifestyle-based prevention strategies as a critical component of post-pandemic metabolic health.

Common manifestations such as chronic fatigue, reduced exercise tolerance, sleep disturbances, and mood disorders not only impair quality of life but may also compound underlying metabolic imbalances. Emerging evidence points to mitochondrial dysfunction as a possible contributor to these lingering symptoms, providing a mechanistic link between long COVID and the metabolic complications discussed above. This overlap further supports the therapeutic value of lifestyle interventions, particularly structured physical activity, nutritional optimization, and stress reduction, as a means to restore metabolic balance and promote recovery in individuals experiencing prolonged post-COVID sequelae [95].

### 5.1. Dietary Recommendations in Post-COVID Recovery

A multifaceted approach combining lifestyle modification, targeted nutritional strategies, and select pharmacologic agents may be beneficial to support recovery. Nutrients and antioxidants such as coenzyme Q10, N-acetylcysteine, alpha-lipoic acid, and L-carnitine have been proposed to mitigate mitochondrial oxidative stress and restore energy metabolism. Moreover, boosting NAD⁺ levels, a key coenzyme in mitochondrial function, through supplementation with nicotinamide riboside (NR) or nicotinamide mononucleotide (NMN) may further enhance cellular repair. Dietary interventions rich in omega-3 fatty acids, B vitamins, and minerals like magnesium also hold promise in alleviating mitochondrial-related symptoms and improving overall recovery [95].

In parallel with the need for targeted nutritional support to restore mitochondrial function, the overall diet quality plays a critical role in shaping long-term recovery and resilience. Diets high in added sugars, saturated fats, and ultra-processed carbohydrates, characteristic of the Western dietary pattern, have been strongly associated with increased incidence of diabetes, obesity, and hypertension and greater susceptibility to COVID-19 infection and its severe outcomes through impaired adaptive immunity [96]. When combined with COVID-19-induced systemic inflammation, these poor dietary habits may further contribute to lasting health complications, including a heightened risk for neuroinflammatory conditions such as neurodegenerative diseases and dementia. Therefore, ensuring access to and promoting the adoption of nutrient-rich, anti-inflammatory diets should be considered a public health priority to reduce metabolic disease risk and mitigate the long-term consequences of COVID-19 recovery [97].

Building on the importance of dietary quality in post-COVID metabolic health, adopting a plant-forward diet rich in whole, fiber-dense foods has been consistently shown to reduce the risk of T2DM and other chronic diseases. Diets emphasizing vegetables, legumes, nuts, seeds, whole grains, and fruits while minimizing added sugars, saturated fats, and processed foods are protective and therapeutic in managing T2DM and reducing the risk of macrovascular and microvascular complications. Meeting recommended fiber intake thresholds (over 25 g/day for women and 38 g/day for men) is critical in glycemic control, insulin sensitivity, and systemic inflammation. In contrast, excess caloric intake from high-sugar and high-fat foods contributes to adiposity, which increases the likelihood of non-insulin-dependent diabetes. However, not all fats carry the same risk. The inclusion of healthy fats, particularly marine-derived omega-3 fatty acids, such as those found in fish, has been shown to have a protective effect against the development of T2DM [90]. In the context of long COVID recovery, this dietary pattern not only supports mitochondrial health and reduces inflammation but also offers a sustainable foundation for restoring metabolic balance and resilience.

### 5.2. Physical Activity and Exercise 

Exercise and targeted rehabilitation protocols tailored for individuals with long COVID-19 have shown promise in enhancing mitochondrial biogenesis by stimulating mitochondrial division and growth, thereby alleviating many of the persistent symptoms associated with the condition [95]. Inactivity, by contrast, has been linked to a significantly higher relative risk of COVID-19 hospitalization [93], and on a global scale, physical inactivity is responsible for approximately 7.2% of type 2 diabetes cases and 9.4% of all-cause mortality [98,99]. Emerging evidence also suggests that even replacing sedentary time with light-intensity physical activity can lead to meaningful improvements in diabetes risk markers, reinforcing the importance of movement at all levels of fitness [100]. Regular physical activity is a cornerstone for improving glycemic control, enhancing insulin sensitivity, reducing low-grade inflammation, optimizing blood lipid profiles, and improving vascular function and body composition. Both aerobic and resistance exercises have demonstrated benefits in lowering blood glucose and HbA1c levels, improving systolic blood pressure, and enhancing liver and pancreatic function, all of which are critical for both long COVID recovery and diabetes prevention and management [101].

Regular physical activity also plays a crucial role in enhancing immune function, a key factor in preventing and recovering from viral illnesses like COVID-19. Physical exercise modulates immune responses by improving the release of pro- and anti-inflammatory cytokines, enhancing lymphocyte circulation, and promoting immune cell recruitment. These changes contribute to reduced infection rates, milder symptoms, and lower mortality in viral diseases, including COVID-19 [102,103]. Exercise promotes a favorable cytokine milieu by enhancing the production of anti-inflammatory mediators like IL-10 while regulating pro-inflammatory cytokines such as IL-6, both through acute bouts and long-term training adaptations [104]. It also increases lymphocyte circulation, especially natural killer (NK) cells and T-lymphocytes, thereby enhancing immune surveillance and early pathogen detection [105]. In addition, acute physical activity mobilizes neutrophils and monocytes, leading to improved immune cell recruitment and accelerated pathogen clearance [106]. These immunological benefits are reflected in clinical outcomes: meta-analyses report a 31% reduction in respiratory infections among physically active individuals [107], with COVID-19-specific studies showing 36% lower hospitalization and 43% reduced ICU admission rates in regular exercisers [108]. Furthermore, physically inactive COVID-19 patients had a 2.5-fold higher mortality risk compared to their active counterparts [108]. Mechanistically, these outcomes are attributed to exercise-induced modulation of inflammation, immune cell kinetics, and tissue resilience, as discussed by Jimenez-Pavon et al. [109]. Collectively, these findings support the integration of regular exercise into preventive strategies for viral illness, highlighting its critical role in enhancing immune competence and reducing disease severity.

Beyond its impact on immune resilience, consistent engagement in physical activity significantly lowers the risk of all forms of diabetes and cardiovascular disease, offering widespread health benefits across all age groups and genders. To achieve meaningful health outcomes, current guidelines recommend at least 150 minutes per week of moderate to vigorous physical activity [90]. Exceeding this threshold can further enhance outcomes, including reductions in body weight and BMI, improved glycemic control, and lowered HbA1c levels, all of which support cardiovascular health and long-term glycemic homeostasis [110]. In light of this evidence, promoting regular physical activity during and beyond the COVID-19 pandemic emerges as a public health priority to not only enhance diabetes management but also curb the growing global burden of both COVID-19 and metabolic disease.

Complementing these insights, a study by Antonarelli and Fogante [111] investigated the impact of COVID-19 on muscle mass and the development of sarcopenia in critically ill patients. Using CT scans from 112 ICU patients requiring mechanical ventilation, the researchers measured the pectoralis muscle area (PMA) and density (PMD) as indicators of muscle mass. Findings from this study demonstrated that reduced muscle mass was significantly associated with poorer clinical outcomes, including prolonged ICU stays and a higher likelihood of extubation failure—the inability to maintain spontaneous breathing following ventilator removal. These observations underscore the role of COVID-19 in accelerating sarcopenia, a degenerative loss of skeletal muscle mass and function, particularly in those with severe disease. Routine evaluation of muscle status through imaging already performed for lung assessment may serve as a valuable, non-invasive tool to identify patients at risk and inform targeted interventions as part of comprehensive care in COVID-19 recovery.

Altogether, these findings highlight the critical role of physical activity in improving metabolic and immune outcomes and preserving muscle integrity during and after COVID-19 illness. As such, incorporating exercise and routine muscle assessment into post-COVID care plans is essential for supporting full recovery, reducing long-term complications, and preventing diabetes progression.

### 5.3. Weight Management and Metabolic Health

Weight gain is a well-established risk factor for the development of diabetes and cardiovascular disease (CVD), and its prevalence notably increased during the early stages of the COVID-19 pandemic. Emerging evidence now indicates that weight gain following COVID-19 infection independently elevates the risk of CVD, even among individuals without prior obesity. Notably, non-obese individuals who gained weight post-infection demonstrated a greater CVD risk than those who maintained their weight, while those with obesity also experienced heightened risk. These findings underscore the urgent need for proactive weight management in the post-COVID landscape as a strategy to prevent long-term cardiometabolic complications. 

While prior research has established associations between COVID-19, obesity, and adverse cardiovascular outcomes, recent studies highlight weight gain itself, regardless of baseline obesity, as a key independent contributor. The mechanisms underlying this elevated risk are thought to involve systemic inflammation, endothelial dysfunction, and disruptions in the ACE2 pathway, all of which may be exacerbated by post-infection metabolic changes [112]. Effective management of long COVID must, therefore, include comprehensive strategies to address modifiable cardiometabolic risk factors. This includes closely monitoring and controlling blood pressure, lipid levels, and body weight and promoting lifestyle interventions such as physical activity, nutritional optimization, and smoking cessation. 

### 5.4. The Role of Stress, Steroids, and Sleep Disruption in Post-COVID Diabetes Risk

Stress hyperglycemia, a transient elevation in blood glucose during acute illness, is a well-documented phenomenon in hospitalized patients and prominently observed in COVID-19 [113]. It arises from the surge of counterregulatory hormones and pro-inflammatory cytokines triggered by the body’s stress response to severe infection. While this hyperglycemic state is considered a temporary and adaptive physiological response, mounting evidence suggests that it may also serve as a precursor to persistent glucose dysregulation, particularly in individuals with underlying metabolic vulnerability. As such, stress hyperglycemia not only reflects acute illness severity but may act as a mechanistic bridge linking COVID-19 to the development of new-onset diabetes [114]. Beyond the direct metabolic consequences, the broader physical and psychological health of individuals with diabetes has been further compromised by the socioeconomic stressors of the pandemic. Lockdowns, financial hardship, and bereavement have exacerbated mental health challenges, with rising levels of anxiety and distress particularly evident in patients managing chronic diseases like diabetes [115]. These intersecting stressors, physiological and psychosocial, collectively amplify the long-term burden of diabetes in the post-COVID era.

COVID-19 and other severe infections can trigger hyperglycemia through a surge in stress hormones such as cortisol and catecholamines, which stimulate hepatic glucose production. In severe COVID-19, the widespread use of corticosteroids further amplifies this effect by promoting gluconeogenesis, accelerating the breakdown of lipids and proteins, and inducing insulin resistance. This steroid-induced insulin resistance is primarily mediated through disruption of the GLUT-4 glucose transporter in skeletal muscle, substantially impairing cellular glucose uptake. While individuals with healthy metabolic function may compensate through increased insulin secretion, those with predisposing factors such as obesity or baseline insulin resistance face a significantly heightened risk of sustained hyperglycemia and the onset of DM due to the combined metabolic strain of infection and treatment [116]. Growing evidence highlights a strong association between sleep disturbances and impaired glucose regulation [117]. Sleep deprivation, prolonged sleep duration, and poor sleep quality have all been linked to dysregulated glucose homeostasis and increased diabetes risk [118,119]. These circadian rhythm and metabolic balance disruptions further exacerbate vulnerability to COVID-19-related glycemic complications, underscoring the need to address sleep health as part of comprehensive diabetes prevention and management strategies in the post-COVID era.

**Figure 4:**
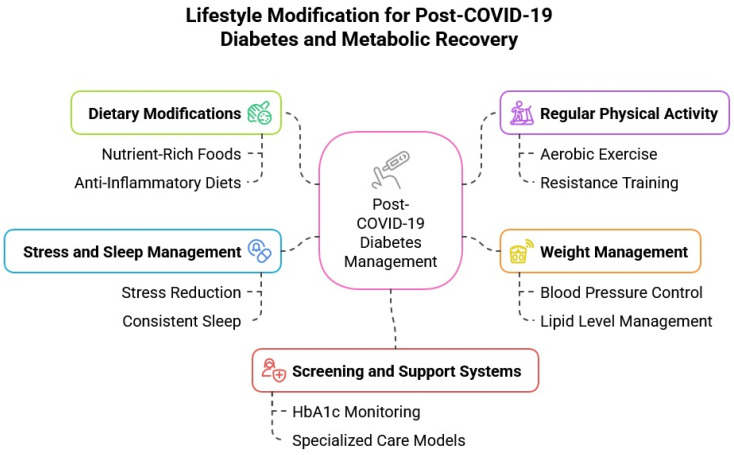
Lifestyle strategies for post-COVID-19 diabetes management. This figure outlines key lifestyle interventions to support diabetes control and metabolic recovery following COVID-19. Core components include dietary modifications, regular physical activity, weight management, stress and sleep regulation, and structured screening and support systems. These multidomain strategies aim to optimize glycemic control, reduce cardiometabolic risk, and improve long-term outcomes in post-COVID-19 patients.

The bidirectional relationship between sleep and metabolic health is well established, with sleep disturbances contributing to and resulting from conditions such as obesity and DM. Sleep deprivation disrupts key hormonal pathways, including dysregulation of ghrelin and leptin (which control hunger and satiety) and elevation of cortisol, the primary stress hormone. These imbalances impair impulse control, promote overeating, and drive insulin resistance and weight gain. Conversely, obesity increases the risk of sleep-related disorders like obstructive sleep apnea, perpetuating a vicious cycle of metabolic dysfunction. The COVID-19 pandemic has intensified these interconnections. Widespread stress, lifestyle disruption, and reduced sleep quality have further fueled the rise in metabolic comorbidities, including T2DM. Addressing both sleep and weight is, therefore, essential for interrupting this harmful feedback loop and reducing the long-term burden of cardiometabolic disease [120]. Meta-analyses confirm a U-shaped relationship between sleep duration and T2DM risk: sleeping fewer than 7 h per night raises diabetes risk by 9% for each hour of lost sleep, while sleeping more than 8–9 h increases risk by 14% for every additional hour. These findings reinforce the importance of maintaining moderate, consistent sleep durations to preserve metabolic health and prevent diabetes progression [121]. Figure 4 outlines the key components of a comprehensive lifestyle-based approach to post-COVID-19 diabetes management, highlighting the roles of diet, physical activity, sleep, weight control, and ongoing monitoring.

### 5.5. Screening, Surveillance, and Support for At-Risk Populations

There is clear and growing evidence that comprehensive care and ongoing support for individuals with diabetes significantly improve overall health outcomes and reduce the risk of complications. In the wake of the COVID-19 pandemic, the role of the nurse case manager specializing in lifestyle medicine has become a viable and effective model for optimizing the management of patients with type 2 diabetes [122].

A recent study highlights that adults over 18 are at increased risk for developing new-onset diabetes, hypertension, and dyslipidemia in the months following COVID-19 infection, presenting with persistent disruptions in glucose metabolism, blood pressure regulation, and lipid profiles. These alterations should be formally recognized as manifestations of post-COVID syndrome, reinforcing the urgent need for evidence-based clinical guidelines to support early identification and management [123]. Key predictors of new-onset diabetes in non-diabetics include a positive family history, elevated BMI, higher cumulative steroid exposure, and prolonged illness duration. Consequently, routine HbA1c monitoring is recommended at follow-up, particularly for individuals recovering from severe COVID-19, which itself should be considered a potential independent risk factor for diabetes development [124].

Moreover, indirect consequences such as fatigue, depression, chronic musculoskeletal pain, and sleep disturbances can exacerbate metabolic dysregulation in the post-COVID setting. The physical and emotional burden of the disease, often compounded by social isolation, has contributed to a notable rise in anxiety and depressive symptoms, especially in patients recovering from ICU-level care. Given their immunocompromised state, patients with diabetes also warrant prioritization for vaccination to mitigate the risk of reinfection and its sequelae [32]. Early detection remains critical. The American Diabetes Association (ADA) recommends regular glucose screening for high-risk populations, including adults over 45 years old, individuals who are overweight or obese, those with prediabetes or a sedentary lifestyle, and anyone with a family history of diabetes. Screening tools such as the oral glucose tolerance test, fasting and postprandial glucose measurements, 1-h glucose testing, waist circumference assessments, and novel technologies like EZSCAN offer standardized and globally accepted methods for early identification of DM. Implementing these strategies in clinical practice is essential for timely diagnosis, effective intervention, and prevention of long-term COVID-19 complications [90].

### 5.6. Recommended Clinical Approach for Managing Post-COVID Diabetes

In light of these emerging risks and evolving clinical patterns, there is a pressing need to operationalize this knowledge into structured care pathways that support early intervention and continuity of care. Primary care providers, endocrinologists, and multidisciplinary teams must work in coordination to ensure that individuals at risk of post-COVID metabolic complications are promptly identified, appropriately screened, and systematically followed [125]. The following clinical framework outlines a stepwise approach, from acute care to long-term monitoring, that integrates risk assessment, diagnostic evaluation, and evidence-based management strategies for patients with or at risk for post-COVID diabetes.

First, early identification and follow-up of individuals at risk for post-COVID diabetes is critical for timely intervention. Risk stratification should begin during acute illness or hospital discharge, focusing on patients hospitalized or admitted to the ICU for COVID-19 and those with underlying metabolic conditions such as obesity, metabolic syndrome, pre-diabetes, a history of gestational diabetes, or polycystic ovary syndrome [94]. Prolonged use of systemic corticosteroids or symptoms of hyperglycemia (e.g., polyuria, polydipsia, unexplained weight loss) should also prompt closer monitoring [126]. If frank ketosis or a random plasma glucose ≥ 300 mg/dL is observed, urgent endocrinology referral is recommended. Between 4 and 12 weeks post-infection, all at-risk or symptomatic patients should undergo baseline screening with HbA1c and fasting plasma glucose (FPG); a 2-h oral glucose tolerance test (OGTT) may be added for higher-risk individuals. Diagnosis should follow the American Diabetes Association (ADA) criteria, and atypical presentations should prompt further evaluation, including autoantibodies and C-peptide levels, to assess for autoimmune or atypical forms of diabetes [127].

For those with normal screening results, glycemic surveillance should be repeated at 6 and 12 months post-infection to identify delayed onset. Individuals with pre-diabetes should begin lifestyle interventions and may be considered for metformin therapy based on age and BMI [128]. Continuous or flash glucose monitoring can be useful in cases of glycemic variability, steroid tapering, or reduced kidney function. In patients with confirmed post-COVID diabetes, management should include comprehensive lifestyle counseling, first-line metformin, and early initiation of GLP-1 receptor agonists or SGLT2 inhibitors in those with high cardiovascular or renal risk [129,130]. Vaccination status should be updated, and endocrinology should assess for atypical progression or complications. Long-term monitoring should include quarterly HbA1c checks until targets are met, followed by biannual assessments, along with regular evaluations of blood pressure, lipids, and BMI. Endocrinology referrals are warranted for persistent hyperglycemia, escalating insulin needs, or recurrent hypoglycemia [131]. Table 2 summarizes this recommended stepwise approach, outlining key actions across each stage of post-COVID diabetes identification and management.

## 6. Conclusions

**Figure 5:**
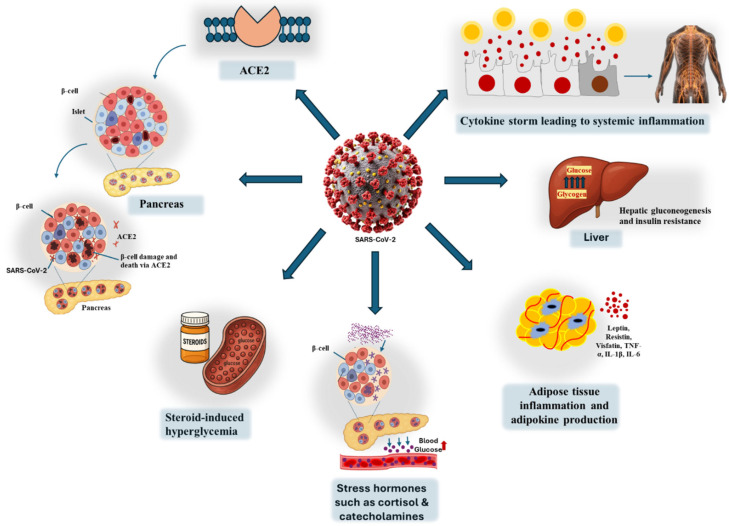
Pathways linking COVID-19 to diabetes. SARS-CoV-2 enters cells via ACE2, affecting multiple organs involved in glucose metabolism. In the pancreas, viral entry into β-cells causes cell death and reduced insulin secretion. A systemic cytokine storm promotes widespread inflammation and insulin resistance. In the liver, inflammation enhances gluconeogenesis and disrupts insulin signaling. Adipose tissue releases pro-inflammatory adipokines, further impairing insulin sensitivity. Stress hormones (cortisol, catecholamines) and steroid treatment also contribute to hyperglycemia through their effects on insulin secretion and glucose metabolism.

The COVID-19 pandemic has revealed a complex and concerning intersection between viral infection and long-term metabolic health, particularly the onset and progression of DM (summarized in Figure 5). Compelling evidence from global cohort studies, mechanistic investigations, and emerging case reports underscores the virus’s ability to trigger both type 1 and type 2 diabetes through direct pancreatic injury, systemic inflammation, immune dysregulation, and therapeutic side effects. The metabolic consequences of SARS-CoV-2 infection persist well beyond the acute phase and are further amplified by lifestyle disruptions, stress, and comorbidities, especially in already vulnerable populations. The pandemic has underscored the urgent need for integrated strategies focused on prevention, early detection, and long-term management of post-COVID diabetes. Lifestyle interventions, including nutritional optimization, physical activity, stress reduction, and sleep hygiene, emerge as critical tools to counteract the metabolic fallout of COVID-19. Moreover, regular screening for diabetes in high-risk individuals, particularly those recovering from moderate to severe infection, is essential. Recognizing post-COVID diabetes as a key component of long COVID will enable more effective clinical surveillance and targeted therapies. As the world transitions into a post-pandemic era, addressing the long-lasting metabolic sequelae of COVID-19 must become a global health priority to reduce the rising burden of diabetes and its complications.

## Figures and Tables

**Table 1:** Summary of studies investigating new-onset diabetes mellitus after COVID-19 recovery.

Reference	Study Type	Sample Size	Follow-Up Duration	Key Findings	Risk Metrics
Chen, M. (2021) [18]	Longitudinal	64	6 months	Insulin resistance increased in patients without prior DM; elevated FBG at 6 months	N/A
Ayoubkhani, D. (2021) [19]	Cohort Study	47,780	140 days	Higher rates of new-onset DM in hospitalized patients compared to controls.	HR 2.47 (acute phase), HR 1.60 (4 months post)
Reges, O. (2023) [20]	Retrospective Cohort Study	157,936	18 months	Acute and post-acute COVID-19 were associated with new DM in severe hospitalized patients	HR 2.47 (acute phase) HR 1.60 (post-infection)
Xie, Y. (2022) [21]	Cohort Study	181,280	352 days	Increased risk of new-onset DM and anti-hyperglycemic therapy initiation post-COVID.	HR 1.40 (new-onset DM), HR 1.85 (therapy initiation)
Wander, P.L. (2022) [22]	Retrospective Cohort study	128,255	120 days	Significant association between COVID-19 and increased DM risk, particularly in men	OR 1.75 (men at 120 days)
Banerjee, M. (2022) [23]	Meta-Analysis	5.7 million	28 days	59% increased risk of DM in the post-acute phase of COVID-19	HR 1.59 (overall), HR 1.52 (hospitalized), HR 1.22 (mild)
Jayaseelan, V. (2022) [24]	Prospective Cohort	724	3 months	Increased risk of DM in patients with moderate to severe COVID-19	RR 2.83 (moderate/severe cases)
Kendall, E.K. (2021) [7]	Retrospective Cohort	1 million	6 months	Significant increase in new-onset T1DM in children post-COVID-19	HR 1.96 (1 month), HR 2.10 (3 months), HR 1.83 (6 months)
Birabaharan, M. (2022) [11]	Retrospective Analysis	600,055	180 days	Higher incidence of T2DM in COVID-19 patients compared to influenza	RR 1.54 (mild), RR 1.46 (moderate/severe)
Herczeg, V. (2022) [6]	Retrospective Analysis	26	3 months	The newly diagnosed T1DM patients had a higher rate of anti-SARS-CoV-2	OR 3.74
Tazare, J. (2022) [13]	Cohort Study	77,347	4 months	COVID-19 patients had higher risk of T2DM	HR 1.46
Rathmann, W. (2022) [15]	Retrospective Cohort Study	35,865	30 days	COVID-19 confers an increased risk for type 2 diabetes	OR 1.28

**Table 2:** Clinical pathways for screening, monitoring, and managing post-COVID diabetes.

Stage	Recommended Actions in Primary Care	Referral Guidelines for Endocrinology
Risk stratification (during or at discharge)	Identify adults or children with any of the following: -COVID-19-related hospitalization/ICU stay -Pre-existing obesity, metabolic syndrome, gestational diabetes, PCOS, or pre-diabetes -Prolonged systemic steroid use for COVID-19 -Symptoms of hyperglycemia (polyuria, polydipsia, weight loss)	-Immediate referral if ketosis or marked hyperglycemia (random glucose ≥ 300 mg/dL) is detected
Baseline screening (4–12 weeks post-infection)	-Order HbA1c + fasting plasma glucose (FPG). -In high-risk or symptomatic patients, add a 2-h OGTT. -Diagnose using ADA cut-offs (A1c ≥ 6.5%, FPG ≥ 126 mg/dL, 2-h PG ≥ 200 mg/dL, or random PG ≥ 200 mg/dL + symptoms).	-Clarify diabetes type (autoantibodies/C-peptide if T1DM suspected). -Assess for acute complications (DKA, HHS).
Follow-up surveillance	-Normal results: repeat A1c or FPG at 6 months and 12 months. -Prediabetes: lifestyle program ± metformin (if BMI ≥ 35 kg/m^2^ or age < 60 y), retest every 3–6 months.	-Consider CGM or flash monitoring if glucose variability is suspected, CKD stage ≥ 3, or steroid taper is ongoing.
Management of confirmed post-COVID diabetes	-Initiate lifestyle therapy (medical nutrition, weight-loss targets ≥ 5–7%). -Start metformin unless contraindicated; if A1c > 8%, add GLP-1 RA or SGLT2-i early for cardio-renal benefit. -Update vaccinations (COVID-19 boosters, pneumococcal, influenza)	-Optimize regimen for cardio-renal risk; screen for pancreatic autoimmunity if insulin requirements rise rapidly. -Arrange complication screening (retina, kidneys, feet) at diagnosis, then annually.
Long-term monitoring	-A1c every 3 months until goal (<7% for most), then every 6 months. -Blood pressure, lipids, and BMI at each visit.	-Evaluate persistent or atypical hyperglycemia, insulin dependence, or severe hypoglycemia.

ADA, American Diabetes Association; CGM, continuous glucose monitoring; CKD, chronic kidney disease; DKA, diabetic ketoacidosis; FPG, fasting plasma glucose; GLP-1 RA, glucagon-like peptide-1 receptor agonist; HHS, hyperosmolar hyperglycemic state; ICU, intensive care unit; OGTT, oral glucose tolerance test; PCOS, polycystic ovary syndrome; PG, plasma glucose; SGLT2i, sodium–glucose cotransporter 2 inhibitor.
